# Pathological patterns of mesangioproliferative glomerulonephritis seen at a tertiary care center

**Published:** 2014-07-01

**Authors:** Ghadeer A. Mokhtar, Sawsan Jalalah, Shabnum Sultana

**Affiliations:** ^1^Department of Pathology, Faculty of Medicine, King Abdulaziz University, Jeddah, Saudi Arabia

**Keywords:** Mesangioproliferative glomerulonephritis, IgM nephropathy, IgA nephropathy

## Abstract

**Background:** Mesangioproliferative glomerulonephritis (MesPGN) is a common morphological pattern that encompasses several groups of renal diseases including IgA nephropathy (IgAN), IgM nephropathy (IgMN), lupus nephritis (LN), C1q nephropathy (C1qN) and other entities.

**Objectives:** The aim of this study was to analyze the pathological findings and the clinical features of cases of MesPGN seen at the king Abdulaziz University, in Saudi Arabia.

**Patients and Methods:** A total of 750 percutaneous native renal biopsies were seen at our institution from January 2000 to December 2011. All the cases diagnosed as MesPGN on light microscopy (LM) were retrieved from the archives of pathology. The pathological features and the clinical data of these cases were reviewed. The clinical data was available for 80 cases only.

**Results:** A total of 103 cases (14%) met the inclusion criteria for the diagnosis of MesPGN. The most common diagnostic entity was IgMN (46.6%) followed by IgAN (30%) along with few cases of class II LN, C1qN, minimal change disease (MCD), Alport’s syndrome, focal segmental glomerulosclerosis (FSGS), thin basement membrane disease (TBMD), and fibrillary glomerulonephritis. The most common clinical presentation was nephrotic syndrome seen in 71% of 80 cases, followed by hematuria (14%). Histologically, focal mesangial proliferation was seen in 62% while diffuse proliferation was seen in 38% of the cases.

**Conclusion:** Mesangioproliferative glomerulonephritis is an important cause of nephrotic syndrome in young adults in the western region of Saudi Arabia. Future studies from the region are needed to elucidate the clinical relevance of mesangial cell proliferation to the end stage kidney diseases.

**Implication for health policy/practice/research/medical education:**

Mesangioproliferative glomerulonephritis is an important cause of nephrotic syndrome in young adults in the western region of Saudi Arabia. Future studies from the region are needed to elucidate the clinical relevance of mesangial cell proliferation to the end stage kidney diseases.

## Introduction

Mesangial cell proliferation and mesangial matrix accumulation are common features in various glomerular disorders. It is well established that mesangioproliferative glomerulonephritis (MesPGN) is a distinct glomerular response pattern characterized by diffuse or focal increase in the number of mesangial cells and expansion of the extracellular matrix in the glomerular mesangium with or without immunoglobulin or complement deposition (1).These features are seen in several disease entities like IgA nephropathy (IgAN) (2), IgM nephropathy (IgMN) (3), systemic lupus erythematous (SLE), Alport’s syndrome, resolving phase of post infectious glomerulonephritis and complement nephropathy (4).

Most cases of MesPGN are caused by IgAN where IgA immune-complex deposits are identified in the glomeruli (5). In general, IgAN has been known to be the most common type of glomerulonephritis (GN) in most countries representing 15-45% of all cases of GN (5,6).

## Objectives

The aim of this study was to analyze cases of MesPGN in the western region of Saudi Arabia, with regard to the clinical presentation and the pathological features, and to compare our data with results obtained from other regions of the world.

## Patients and Methods

### Study patients

A total of 750 percutaneous renal biopsies were seen at our institution between the periods of January 2000 to December 2011. Patients or biopsies were referred from our hospital and also from different hospitals in the region. The pathological reports were reviewed to select all the cases that met the pathological criteria for diagnosing MesPGN. The clinical data (clinical presentation at the onset of the disease, presence or absence of hematuria, level of blood pressure and renal function status at the time of clinical presentation) were obtained from the clinical records. The slides of these cases were also retrieved and reviewed. All renal biopsies were examined by LM, immunofluorescence (IF) and electron microscopy (EM). The renal tissue in each case was divided between these three studies and the frozen renal tissue sections were immunolabelled with FITC-conjugated antibodies against IgG, IgA, IgM, C3, C4, C1q, kappa, lambda and fibrinogen.

### Clinical features

Nephrotic syndrome (NS) was defined as proteinuria of 3.5 g/24 h. Hematuria was defined as 3 or more erythrocytes/high-power field (HPF) of urinary sediment. Hypertension was defined as systolic or diastolic blood pressure above the 95^th^ centile for age, gender and height. Renal insufficiency was defined by a calculated creatinine clearance below normal values for patient age and gender.

### Pathological diagnosis

**Figure 1:**
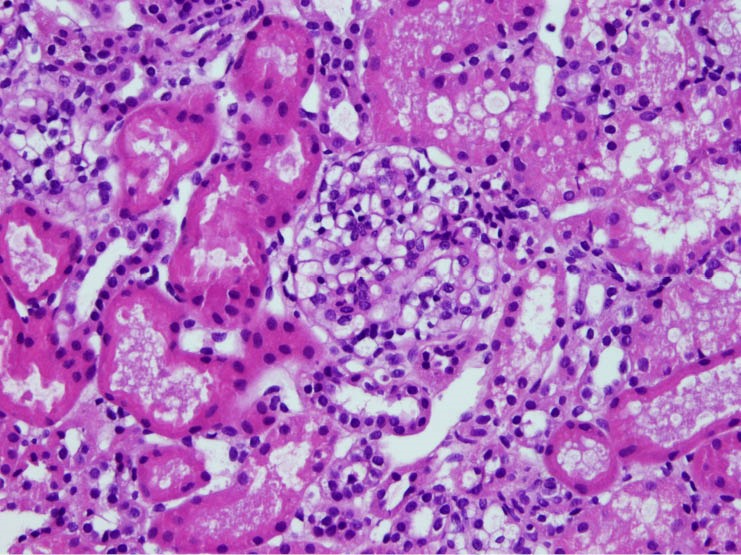
A glomerulus showing segmental mesangial proliferation and mesangial matrix expansion (H&E, 200X)

**Figure 2:**
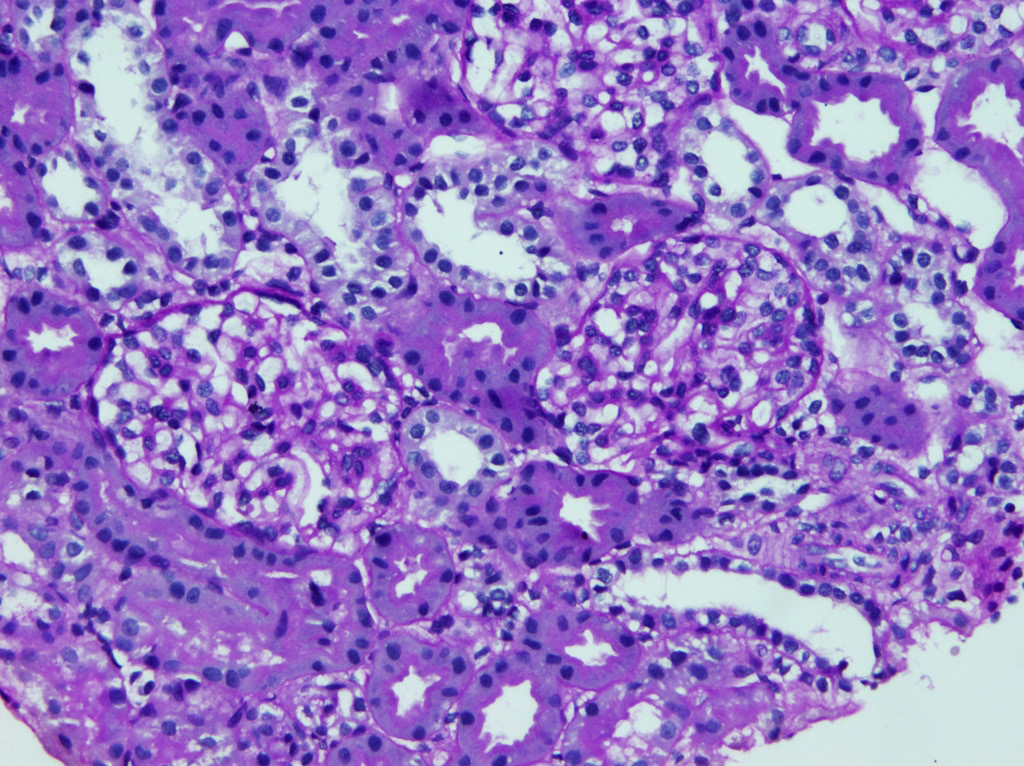
Two glomeruli exhibiting segmental mesangial cells proliferation and matrix expansion (PAS, 200x)

**Figure 3:**
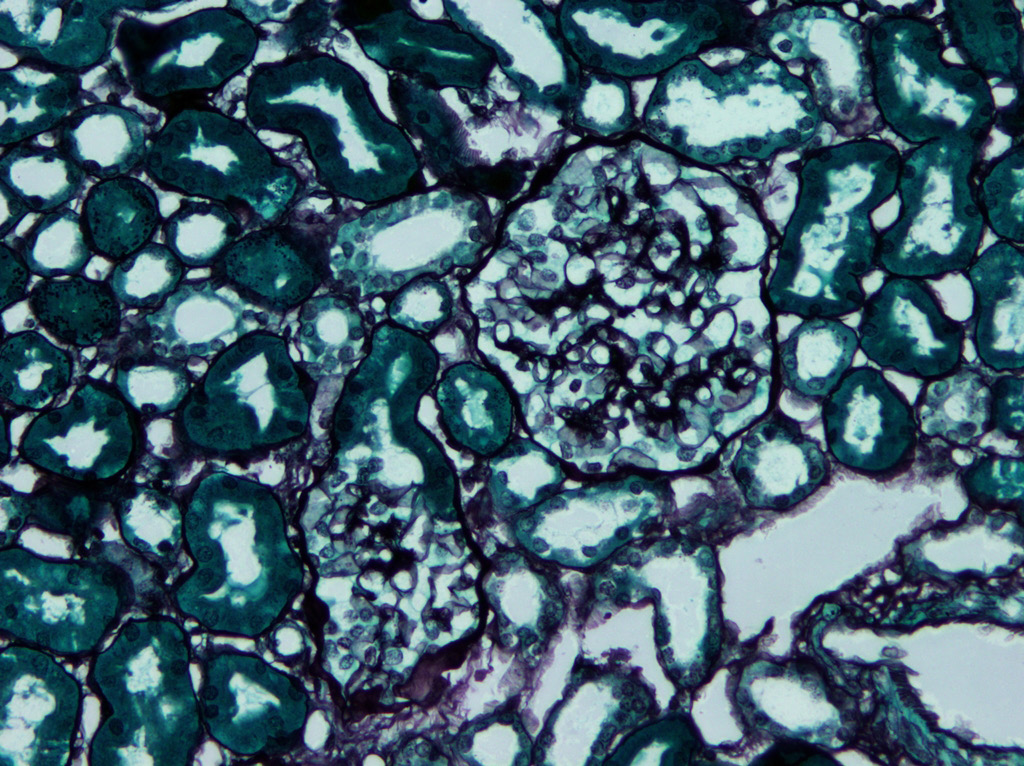
Mesangial matrix expansion in two glomeruli highlighted by silver special stain (JMS, 200X)

The biopsies were classified as MesPGN when the glomeruli showed mesangial hypercellularity, defined as presence of four or more cells per mesangium with or without mesangial matrix expansion and immune complex deposits (1). If the mesangial hypercellularity affected <50% of the total number of the glomeruli, then it is considered focal MesPGN, whereas if > 50% are involved it is considered diffuse MesPGN (Figures 1, 2 and 3).

The presence of glomerular sclerosis, interstitial fibrosis and inflammation and tubular atrophy were also assessed.

### Ethical issues

1) The research followed the tenets of the Declaration of Helsinki; 2) informed consent was obtained; 3) the research was approved by ethical committee of King Abdulaziz University.

### Statistical analysis

The data was analyzed using the SPSS 18. Continuous data were represented as mean or median while categorical data were presented as percentages. In this study, p<0.05 was considered significant.

## Results

### Clinical features

There were 103 out of 750 (14%) of kidney biopsies that met the pathological criteria for the diagnosis of MesPGN. Of these, 17 cases were referred from other hospitals in the region while the remaining was performed at King Abdulaziz University Hospitals (KAUH). There was an equal distribution of cases between males and females with 52 males and 51 females. The patients’ age ranged from 2 years to 68 years with a mean of 19 and a median of 15 years. The clinical presentation was available for 80 cases (Table 1). Fifty seven cases (71%) presented with NS; of these, 38 cases (66.7%) were IgMN, 9 cases (15.8%) were IgAN, 4 cases (7%) were FSGS, 2 cases (3.5%) were fibrillary GN, 2 cases (3.5%) were C1qN and 2 cases (3.5%) of minimal change disease (MCD). Six cases (8%) presented with non-nephrotic proteinuria. Of these, 5 (83%) were LN and 1 case (17%) was IgAN. Hematuria alone was the clinical presentation in 11 cases (14%); the majority of them, 45% were IgAN (5 cases), in addition, 3 cases (27%) were IgMN, 2 cases (18%) were TBMD and one case (9%) was Alport’s syndrome. Combined hematuria and proteinuria were present in 3 cases (4%), with 2 IgAN and 1 FSGS. Hypertension was present in only 1 case of LN, 2 cases of IgAN and one case of IgMN. One case of IgMN and one case of LN also had an impaired renal function with high creatinine level at the onset of the disease. Skin rash was seen in one case with the clinical and pathological diagnosis of Henoch-Schönlein purpura (HSP).

**Table 1:** Correlation of clinical presentation with the pathological diagnosis in 80 cases of mesangioproliferative glomerulonephritis

**Diagnosis**	**NS**	**Hematuria**	**Proteinuria**	**Hematuria and Proteinuria**	**Hypertension**	**CRF**
IgMN	38(66.7%)	3 (26%)	-	-	-	1 (50%)
IgAN	9 (15.8%)	5 (42%)	1 (20%)	2 (67%)	1 (50%)	-
HSP	-	1 (8%)	-	-	-	-
Lupus nephritis class II	-		5 (80%)		1 (50%)	1 (50%)
Fibrillary GN	2 (3.5%)	-	-	-	-	-
MCD	2 (3.5%)	-	-	-	-	-
Alports syndrome	-	1 (8%)	-	-	-	-
TBMD	-	2 (16%)	-	-	-	-
C1qN	2 (3.5%)	-	-	-	-	-
FSGS	4 (7%)	-	-	1 (33%)	-	-
Total	57	12	6	3	2	2

NS= Nephrotic syndrome, CRF: Chronic renal failure, IgMN= Immunoglobulin M nephropathy, IgAN= Immunoglobulin A nephropathy, HSP= Henoch-Schönlein purpura, Fibrillary GN= Fibrillary glomerulonephritis, MCD= Minimal change disease, FSGS= Focal segmental glomerulosclerosis, TBMD= Thin basement membrane disease.

### Pathological findings

The pathological diagnosis were based on combined LM findings, IF and EM study (Table 2). Forty eight cases were IgMN (46.6%); five cases of these were accompanied by secondary FSGS. Thirty one cases (30%) were IgAN and one case was HSP. There were nine cases of LN class II (8.7%), 5 cases of FSGS (4.7%), 2 cases of fibrillary GN (1.9%), 2 cases of MCD (1.9%), 2 cases of TBMD (1.9%) and one case of Alport’s syndrome (0.97%).

**Table 2:** Frequency of pathological diagnosis in 103 cases of mesangioproliferative glomerulonephritis

**Pathological Diagnosis**	**Number**	**%**
IgMN	48	46.61
IgAN	31	30.09
HSP	1	0.97
Lupus nephritis class II	9	8.74
Fibrillary GN	2	1.94
MCD	2	1.94
FSGS	5	4.86
Alports	1	0.97
TBMD	2	1.94
C1q Nephropathy	2	1.94
Total	103	100

IgMN= Immunoglobulin M nephropathy, IgAN= Immunoglobulin A nephropathy, HSP= Henoch-Schönlein purpura, Fibrillary GN= Fibrillary glomerulonephritis, MCD= Minimal change disease, FSGS= Focal segmental glomerulosclerosis, TBMD= Thin basement membrane disease.

Focal mesangial proliferation was seen in 64 cases (62%) (Table 3), 37 cases (58%) were IgMN. Segmental proliferation was also noted in FSGS, TBMD, Alport’s syndrome, MCD and C1qN. Diffuse mesangial proliferation was noted in 39 cases (38%); 16 of these were IgAN (40%), in addition it was also seen in fibrillary GN and LN.

**Table 3:** Histopathological findings in 103 biopsies of mesangioproliferative glomerulonephritis

**Diagnosis**	**No. of cases**	**Segmental mesnagial proliferation**	**Diffuse mesangial proliferation**
IgMN	48	36 (56.1%)	12 (30.8%)
IgAN	31	15 (23.4%)	16 (41%)
HSP	1	1 (1.7%)	-
Lupus nephritis class II	9	-	9 (23.1%)
Fibrillary GN	2	-	2 (5.1%)
MCD	2	2 (3.1%)	-
FSGS	5	5 (7.8%)	
Alports syndrome	1	1 (1.7%)	-
TBMD	2	2 (3.1%)	-
C1qN	2	2 (3.1%)	-
Total	103	64 (62%)	39 (38%)

IgMN= Immunoglobulin M nephropathy, IgAN= Immunoglobulin A nephropathy, HSP= Henoch-Schönlein purpura, Fibrillary GN= Fibrillary glomerulonephritis, MCD= Minimal change disease, FSGS= Focal segmental glomerulosclerosis, TBMD= Thin basement membrane disease.

Interstitial fibrosis and tubular atrophy were noted in 29 cases (28%), all were the diffuse variant and the majorities were mild.

**Figure 4:**
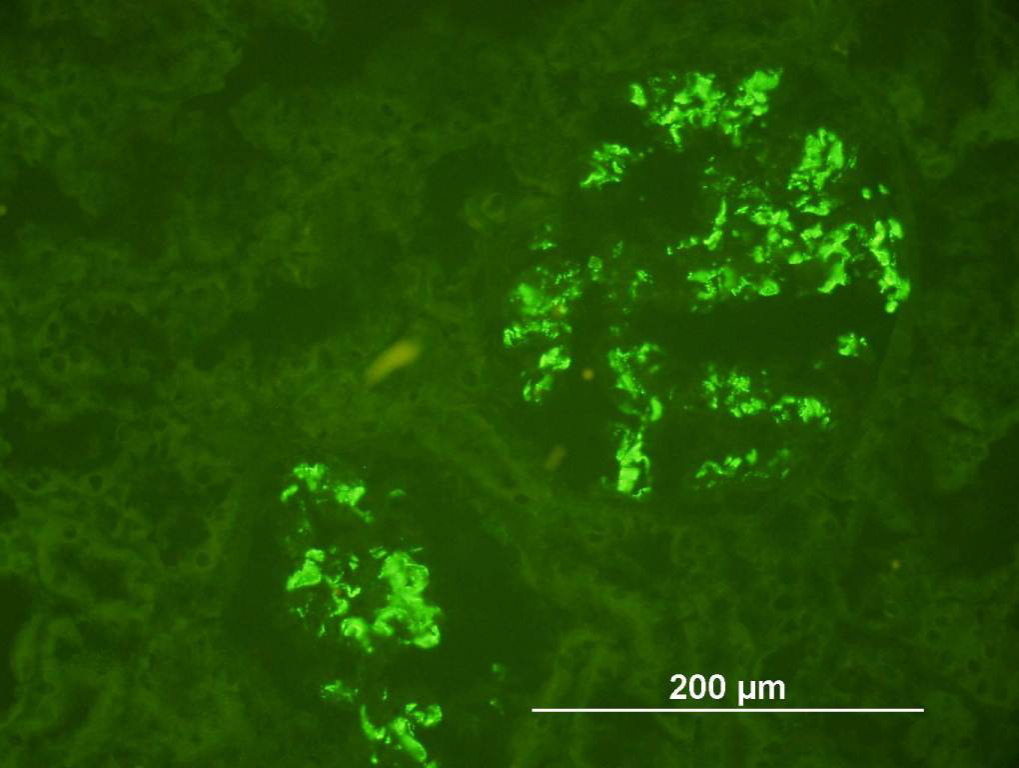
Strong diffuse mesangial staining for IgM (IF, 200x)

By IF study, IgMN showed a strong mesangial granular staining (2+ and 3+) in the mesangial areas for IgM and C3; in addition, IgG was also present in 3 cases (Figure 4). IgAN showed strong granular mesangial staining for IgA and C3; in addition, IgM in 14 cases and IgG in 6 cases. C1qN showed a predominant staining of the mesangium (3+) for C1q along with IgG, IgM and C3. LN showed a full house staining pattern for immunoglobulin and complement in the mesangium. The two cases of fibrillary GN showed mesangial staining for IgG, IgM and IgA along with kappa and lambda light chains. Nonspecific trapping of IgM and C3 was observed by IF in cases of FSGS, while the IF was negative in MCD, TBMD and Alport’s syndrome.

**Figure 5:**
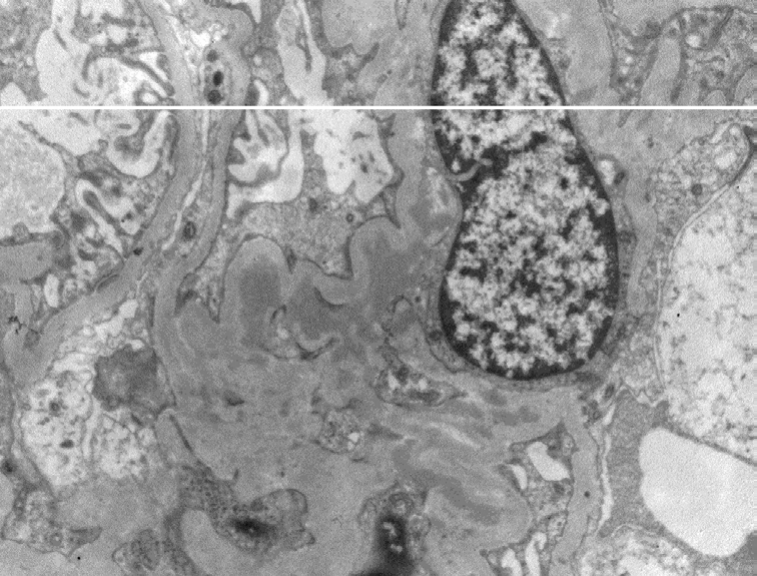
Mesangial electron-dense immune-complex deposit (TEM, 5000X)

By EM examination, mesangial immune-complex deposits were seen in 27 cases of IgMN. All the cases of IgAN showed mesangial and paramesangial deposits, in addition, 7 cases showed subendothelial deposits (Figure 5). All the cases of LN showed mesangial deposits by EM and only one case showed rare subepithelial deposits. C1qN showed small mesangial deposits and fibrillary GN showed mesangial fibrils measuring 14 nm. The case of Alport’s syndrome demonstrated the characteristic EM finding of lamination of the lamina densa along with irregular thin and thick areas of glomerular basement membrane (GBM). TBMD showed marked diffuse thinning of the GBM in comparison to the normal reference for patients’ age.

## Discussion

Mesangioproliferative GN is not uncommon morphological pattern and it has been reported in 10-25% of GN biopsies from different regions of the world (7-10). In our study, it constituted 14% of renal biopsies. The majority of our cases were seen in young age group with a mean age of 19 years with no difference in the prevalence between males and females. This is a younger age group in comparison to other studies in which the majorities of their patients were in the middle age group and showed a male predominance (4,6,7,10).

MesPGN is the most common pathological finding with IgAN, but also seen in a wide variety of GN like IgMN, C1qN, Class II LN, Alport’s syndrome, MCD and FSGS. Worldwide, most cases of MesPGN are caused by IgAN with reported incidence of up to 45% (5,6). However, in our study it is mostly caused by IgMN (48%). This could be explained by high incidence of IgMN in our population, which was reported to constitute 7.8% of 296 GN cases in adults and 14.8% of 169 cases in children from two different studies at our institution (8,9,12).

Focal MesPGN was mostly observed in IgMN while diffuse variant was seen most commonly in IgAN. In a study of 57 cases of MesPGN by Usha et al, focal variant was mostly seen in MCD (7).

NS was the most common clinical presentation (71%) followed by hematuria and combined proteinuria and hematuria. This is very similar to other studies in which nephrotic range proteinuria was seen in up to 82% of their study population (7,10).

The prognosis of MesPGN is excellent with a benign clinical course which was confirmed in two studies with a 3-year renal survival was up to 93% (6,13). However, in a more recent study of 57 patients of non-IgA MesPGN, thirteen of their patients developed chronic kidney disease which was progressed to end-stage kidney disease in three patients (11). There is accumulating evidence that mesangial cell proliferation and mesangial matrix accumulation may contribute to the progression to glomerulosclerosis (14,15). Accordingly it is apparent that mesangial cell proliferation is an important prognostic factor in GN which requires more attention by the pathologist.

## Conclusion

This study presents the clinicopathological correlation of mesangioproliferative disorders in the western region of Saudi Arabia and highlights the importance and the need for longer period of follow up of the cases. Future studies from the region are needed to elucidate the clinical relevance of mesangial cell proliferation to the end stage kidney diseases.

## Authors’ contributions

SS collected and retrieved the cases for review. GM and SJ reviewed the slides and analyzed the data and wrote the manuscript.

## Ethical considerations

Ethical issues (including plagiarism, informed consent, misconduct, double publication and redundancy) have been completely observed by authors.

## Conflict of interests

The authors declared no competing interests.

## Funding/Support

None.

## References

[1] Bohle A, Wehrmann M, Bogenschutz O, Batz C, Vogi W, Schmitt H (1992). The long term prognosis of the primary glomerulonephritis A morphological and clinical analysis. Pathol Res Pract.

[2] Tipu HN, Ahmed TA, Bashir MM (2011). Clinical, histopathological and immunofluorescent findings of IgA nephropathy. Iran J Immunol.

[3] Little MA, Doman A, Gill D, Walshe JJ (2000). Mesangioproliferative glomerulonephritis with IgM deposition Clinical characteristic and outcome. Ren Fail.

[4] Johm M, Lam M, Latham B, Saker B, French AH (2000). Nephrotic syndrome in a patient with IgA deficiency-associated mesangioproliferative glomerulonephritis. Path.

[5] Rychlik I, Andrassy K, Waldherr R, Zuna I, Tesar V, Jancová E (1999). Clinical features and natural history of IgA nephropathy. Ann Med Interne (Paris).

[6] Vikse BE, Bostad L, Aasarød K, Lysebo DE, Iversen BM (2002). Prognosticfactors in mesangioproliferative glomerulonephritis. Nephrol Dial Transplant.

[7] Usha Usha, Kumar S, Singh RG, Tapas S, Prakash J, Garbyal R (2008). Mesangioroliferative glomerulonephritis An important glomerulonephritis in nephrotic syndrome of young adult. Indian Journal of Pathology and Microbiology.

[8] Jalalah SM, Jamal AA (2009). Childhood primary glomerular disease in the Western region of Saudi Arabia. Saudi J Kidney Dis Transpl.

[9] Abdelraheem MB, Ali EM, Mohamed RM, Hassan EG, Abdalla OA, Mekki SO (2010). Pattern of glomerular disease in Sudanese Children: a clincopathological study. Saudi J Kidney Dis Transpl.

[10] Kamib HH, Gharavi AG, Aftimos G, Mahfoud Z, Saad R, Gemayel E (2010). A 5-year survey of biopsy-proven kidney disease in Lebanon: significant variation in prevalence of primary glomerular diseases by age < population structure and consanguinity. Nephrol Dial Transplant.

[11] Waikhom R, Sarkar D, Patil K, Pandey R, Dasgupta S, Jadhav J (2012). Non-IgA mesangioproliferative glomerulonephritis: a benign entity?. Nephrol Dial Transplant.

[12] Jalalah SM (2009). Patterns of primary glomerular diseases among adults in the western region of Saudi Arabia. Saudi J Kidney Dis Transpl.

[13] Owada K, Suzuki H, Katoh T, Watanabe T (2010). Genetical, histological, and clinical characteristics of IgA-negative mesangioproliferative glomerulopathy. Clin Exp Nephrol.

[14] Floege J, Burns MW, Alpers CE, Yoshimura A, Pritzl P, Gordon K (1992). Glomerular cell proliferation and PDGF expression precede glomerulosclerosis in the remnant kidney model. Kidney Int.

[15] Pesce CM, Striker LJ, Peten E, Elliot SJ, Striker GE (1991). Glomerulosclerosis at both early and late stages is associated with increased cell turnover in mice transgenic for growth hormone. Lab Invest.

